# Advanced HIV disease among newly diagnosed clients identified through community- and facility-based HIV testing services in Eswatini: a cross-sectional study

**DOI:** 10.3389/fpubh.2026.1816639

**Published:** 2026-06-11

**Authors:** Samson Haumba, Thokozani Maseko, Fezokuhle Khumalo, Nakekelo Mndzebele, Makhosazana Dlamini, Sylvia Ojoo

**Affiliations:** 1 Center for Global Health Practice and Impact, Georgetown University, Mbabane, Eswatini; 2 Center for Global Health Practice and Impact, Georgetown University, Washington, DC, United States

**Keywords:** Advanced HIV Disease (AHD), CD4, community-based HIV testing, Eswatini, facility-based HIV testing, WHO clinical staging

## Abstract

**Background:**

Despite the Kingdom of Eswatini surpassing the UNAIDS 95–95-95 targets, the persistence of Advanced HIV Disease (AHD) remains a clinical concern. Advanced HIV Disease increases morbidity and mortality. This study evaluated the rate of AHD among newly diagnosed clients aged ≥15 identified through the community-based testing in comparison to the facility-based testing.

**Methods:**

A cross-sectional study was conducted in two groups (community and facility) of individuals newly diagnosed with HIV using secondary program data (October 2022–September 2025) to identify clients with WHO clinical stages 3 and 4 or defining disease which represent advanced HIV disease with severe immunosuppression or with a CD4 count <200 cells/mm^3^. Descriptive and comparative analyses were performed to compare AHD rates between the community and facility group. Bivariate and multivariable logistic regression analyses were utilized to identify associated factors of AHD for each group using STATA 19.5.

**Results:**

The AHD rate was 19.0% (*n* = 1,248) in the facility group and 16.7% (*n* = 88) in the community group, with no statistically significant difference observed between the two groups (*p* > 0.05). In facility group, factors associated with AHD included increase in age (aOR: 1.03 [1.01–1.05]; *p* < 0.001), male sex (aOR: 1.8 [1.5–2.1]; *p* < 0.001), and residing in Manzini (aOR: 1.6 [1.3–1.8]; *p* < 0.001). Conversely, age and sex were not significantly (*p* > 0.05) associated with AHD in community-based testing setting. Delayed ART initiation (8–28 days) was associated with AHD in both groups, that is, community group (aOR: 3.2 [1.9–4.1]; *p* = 0.2) and facility group (aOR: 2.7 [2.1–3.3]; *p* < 0.001), reflecting the clinical complexity of stabilizing AHD prior to ART initiation.

**Conclusion:**

Community testing did not demonstrate an advantage in early diagnosis of HIV in this study, but reached a younger, mobile population than facility testing. Hence community testing can be a complementary strategy for sub populations who often bypass facility-based care as well as used in addressing localized drivers of late presentation. Facility-based testing, meanwhile, remains essential for symptomatic late presenters and people returning to care.

## Introduction

The global effort to end the AIDS epidemic by 2030 has been bolstered by many countries achieving the 95–95-95 UNAIDS targets, yet late presentation to care remains a barrier to achieving zero AIDS-related deaths. Advanced HIV Disease (AHD), defined by the World Health Organization (WHO) as a CD4 cell count < 200 cells/mm^3^ or being in WHO clinical stage 3 or 4, continues to affect a substantial proportion of people living with HIV (PLHIV) at the time of diagnosis (1). WHO clinical stages 3 and 4 represent advanced HIV disease with severe immunosuppression, and Stage 3 involves serious infections (e.g., severe weight loss, chronic diarrhea, pulmonary TB), while Stage 4 indicates AIDS-defining illnesses (e.g., toxoplasmosis, Kaposi sarcoma, esophageal candidiasis). In Children under 5, stage 3 and 4 criteria are specific, but all children <5 with HIV are considered to have AHD. In Sub-Saharan Africa, despite the rapid expansion of Antiretroviral Therapy (ART) and the advent of test and treat, approximately 30–50% of individuals entering care still present with AHD (2). This clinical state is associated with high risks of opportunistic infections, such as tuberculosis and cryptococcal meningitis, leading to increased hospitalization and mortality rates (3–5).

Eswatini has made remarkable progress in controlling its HIV epidemic, becoming one of the first countries to surpass the 95–95–95 targets (6). However, Eswatini still has one of the highest HIV prevalences globally, and the maintenance of these gains requires identifying the remaining undiagnosed individuals. Facility-based testing has been the cornerstone of the national response, but it often relies on passive case-finding, where individuals only receive testing when they are symptomatic or attending the clinic for other health reasons (7, 8). This may lead to a higher concentration of AHD among facility-identified clients, as seen in various regional contexts (9).

To address the limitations of facility-based approaches, Eswatini has scaled up HIV testing Differentiated Service Delivery (DSD) models, including community-based testing. Community-based testing strategies, such as mobile outreach, home-based testing, and index testing, are designed to reach healthy, asymptomatic individuals earlier in their infection by removing geographic and financial barriers to testing (10, 11). The assumption underlying these models is that community identification will lead to earlier diagnosis at higher CD4 counts, thereby reducing the incidence of AHD (12, 13). However, emerging data suggests that even in community settings, certain high-risk groups, particularly men and older adults, continue to present with advanced disease (14, 15).

Gender disparities play a significant role in the timing of HIV diagnosis and the subsequent prevalence of AHD. Research across Southern Africa consistently shows that men are less likely to utilize facility-based services due to social stigmas, masculinity norms, and work-related barriers (16, 17). Consequently, men are frequently diagnosed with AHD at higher rates than women, who often have more contact with health facilities through maternal and child health services (18, 19). Understanding whether community-based models effectively mitigate this gender disparity by identifying men before they reach advanced clinical stages is essential for tailoring national interventions (20).

Furthermore, the implementation of test and treat guidelines has shifted the focus toward immediate ART initiation. While same-day initiation is the standard of care, those presenting with AHD require careful clinical management to avoid Immune Reconstitution Inflammatory Syndrome (IRIS) (21, 22). The transition from community-based diagnosis to facility-based treatment remains a vulnerable point in the care continuum (23). If linkage to care is delayed following a community diagnosis, the clinical benefit of early identification may be lost, potentially allowing patients to progress to AHD before ART is successfully initiated (24, 25).

Despite the strategic importance of these testing modalities, there is a paucity of recent comparative data in Eswatini specifically examining AHD rates between community and facility entry points for individuals aged 15 years and older. Most existing studies focus on general testing yield rather than clinical staging at the time of diagnosis (26, 27). This study aimed to investigate the rate of AHD among clients testing in the community and facility and to identify factors associated with AHD in these settings.

## Methods

### Study design and setting

This was a cross-sectional study conducted in Manzini and Lubombo regions of Eswatini. Manzini is more urban with a population of about 360,245 people, while Lubombo is more rural and has a population of about 215,106 people (28). Using secondary data, we selected two population groups based on the location they received an HIV test, that is, facility or community. In the Manzini region, facility-based testing is conducted in 123 (*n* = 123) health facilities, including four hospitals (with inpatient services), two public health units, and 117 primary health clinics. In the Lubombo region, facility-based testing is conducted in 54 health facilities (*n* = 54), including two hospitals, one rural multi-department health center, one public health unit (providing maternal, child welfare and vaccination services), and 42 primary health clinics. Community-based testing is conducted through mobile outreach, home-based testing, and index testing in both regions.

### Study participants, sample size and sampling

Clinical and demographic data were abstracted from routine patient clinical records for the period between October 2022 and September 2025. Study participants were clients newly diagnosed with HIV, 15 years of age or older, in health facilities and communities in Manzini and Lubombo regions between October 2022 and September 2025. Between October 2022 and September 2025, 533 clients were diagnosed with HIV during community testing (community group), and 6,906 clients were diagnosed during facility-based testing (facility group). We realized, on further scrutiny of the data, that 360 participants (7 in community testing and 353 in facility testing) were not true new positives. They were ART clients who had disengaged in care and used HIV testing to re-engage in care. While we report these participants to highlight the re-engagement behavior of clients (Table 1), we excluded them in comparative and regression analysis, hence the sample sizes used were 526 in the community group and 6,556 in the facility group.

**Table 1:** Description of study participants disaggregated by location of HIV testing.

Parameter	Community	Facility	Total	*P*-value*
	*n* (%)	*n* (%)	*n* (%)	
All participants	533 (7.2)	6,906 (92.8)	7,439 (100.0)	
Age (years)				
15–29	228 (42.8)	2,514 (36.4)	2,742 (36.9)	0.052
30–39	188 (35.3)	2,416 (35.0)	2,604 (35.0)	
40–49	73 (13.7)	1,265 (18.3)	1,338 (18.1)	
50–59	33 (6.2)	529 (7.7)	562 (7.6)	
60+	11 (2.1)	182 (2.6)	193 (2.6)	
Mean (±SD)	32.9 ± 10.2	34.7 ± 10.8	34.5 ± 10.8	0.063
Sex				
Female	346 (64.9)	4,357 (63.1)	4,703 (63.2)	0.237
Male	187 (35.1)	2,549 (36.9)	2,736 (36.8)	
Marital status				
Widowed/divorced/Separated	9 (1.7)	123 (1.8)	132 (1.8)	0.054
Married	62 (11.6)	1,369 (19.8)	1,431 (19.2)	
Single	462 (86.7)	5,413 (78.4)	5,875 (79.9)	
Region				
Lubombo	203 (38.1)	2,520 (36.5)	2,723 (36.6)	0.131
Manzini	330 (61.9)	4,386 (63.5)	4,716 (63.4)	
Smoking				
Yes	10 (1.9)	130 (1.9)	140 (1.9)	0.671
No	523 (98.1)	6,776 (98.1)	7,299 (98.1)	
Alcohol intake				
Yes	30 (5.6)	439 (6.4)	469 (6.3)	0.112
No	503 (94.4)	6,467 (93.6)	6,970 (93.7)	
ART initiation status				
Previously Initiated on ART (not true new positive)	7 (1.3)	353 (5.1)	360 (4.8)	
Same day Initiation (0–7 days)	455 (85.4)	5,937 (86.0)	6,392 (85.9)	0.218
Delayed Initiation (8–28 days)	39 (7.3)	395 (5.7)	434 (5.8)	
Delayed Initiation (>28 days)	29 (5.4)	183 (2.7)	212 (2.9)	
Pending Initiation	3 (0.6)	38 (0.6)	41 (0.6)	

*Compares distribution of participants’ characteristics between Community and Facility testing and excludes participants previously initiated ART.

### Data sources and study variables

Data was extracted from the national electronic medical records system, the Client Management Information System (CMIS), on 28th November 2024. The required variables were filtered and extracted per the study objectives. The variables describe the patient’s sociodemographic and clinical information (Table 1). Age was grouped into 5 categories from 15 to 60+ years. Advanced HIV disease was defined as having a CD4 cell count < 200 cells/mm^3^ or being in WHO clinical stage 3 or 4 or having a WHO stage 3 or 4 defining disease at the time of HIV diagnosis. A clients with any (one or all) of these defining characteristics were classified as having AHD. In cases of discordance, such as a client with a CD4 cell count < 200 cells/mm^3^ and a WHO clinical stage 2, we classified the client based the available documented indicator for AHD.

### Statistical analysis

Extracted data was imported into Stata 19 (College Station, TX) for cleaning and analysis. Descriptive analysis was conducted to summarize key characteristics of the participants in the community and facility groups. Age was analyzed as a continuous variable before being categorized and was summarized using means and standard deviations. Categorical variables were summarized using frequencies and proportions. Key participant characteristics are presented in Table 1, disaggregated by community and facility testing. All participant characteristics obtained for analysis had no missing observations. The outcome variable was AHD and had no missing observations. The outcome variable was a binary variable (AHD: yes/no) generated from the participants CD4 cell count or WHO clinical staging or WHO stage 3 or 4 defining disease. We assessed differences in the distribution of participants between community and facility groups using Chi square for all nominal variables (sex, marital status, region, smoking, and alcohol use), Pearson’s correlation for grouped age, Spearman’s Rho and Fisher’s exact test for ART initiation status, and independent *t*-test to compare means in age. To compare AHD rate between community and facility testing, comparative analyses were conducted, where Chi-square was used for nominal variables (sex, marital status, region, smoking, and alcohol use), Pearson’s correlation for ordinal parametrically distributed variables (grouped age), and Spearman’s correlation was used for non-parametrically distributed variables (ART initiation status). Fisher’s exact was computed and significance level reported in variables with subcategories containing less than five participants (grouped age, marital status, region, smoking, alcohol use, ART initiation status). Significance was determined at *p* < 0.05. Bivariable logistic regression was used to determine the crude odds ratios (cOR), and multivariable logistic regression through forced entry was used to determine the adjusted odds ratios (aOR) for predictors of AHD in each group separately. Secondary to that the programmatic data had fewer variables available for analysis, all variables were assessed for association with AHD and included in multivariable logistic regression modeling. A link test was conducted for each model specification, and each model was well specified (hatsq *p* > 0.05). To test calibration in the developmental sample of each of the model, Hosmer–Lemeshow goodness-of-fit test in 10 subgroups was conducted, and each model was a good fit in predicting the outcome variable, and without overfitting (Facility group: Hosmer–Lemeshow = 6.35, *p* > 0.05, Area under ROC curve = 0.87 and community group: Hosmer–Lemeshow = 7.41, *p* > 0.05, Area under ROC curve = 0.76).

## Results

Table 1 describes the 533 participants in the community group and 6,906 in the facility group. Community-tested participants were slightly younger on average (mean age 32.9 ± 10.2 years) compared to those tested at facilities (34.7 ± 10.8 years), with a higher proportion of the 15–29 years age band in the community (42.8%, *n* = 228) than in the facility (36.4%, *n* = 2,514) setting, but with no evidence of statistical difference (*p* > 0.05). Sex distribution of participants was relatively similar across both groups, with females comprising the majority in both community (64.9%, *n* = 346) and facility (63.1%, *n* = 4,357) settings (*p* > 0.05). A higher proportion of participants identified in the community were single (86.7%, *n* = 462) compared to those identified at facilities (78.4%, *n* = 5,413), whereas the facility setting a higher rate of married participants (facility: 19.8%, *n* = 1,369 vs. community: 11.6%, *n* = 62), but there was no evidence of significant statistical difference in marital status between the groups. The rate of smoking (facility: 1.9%, *n* = 130 vs. community: 1.9%, *n* = 10) and alcohol intake (facility: 6.4%, *n* = 439 vs. community: 5.6%, *n* = 30) were low in both groups (*p* > 0.05). While same-day initiation of ART after HIV testing was high in both groups (community: 85.4%, *n* = 455; vs. facility: 86.0%, *n* = 5,937), community-identified participants had a higher rate of delayed ART initiation (>28 days) at 5.4% (*n* = 29) compared to 2.7% (*n* = 183) for those tested at a facility, but there was no evidence of significant statistical difference in ART initiation status between the groups. Facility testing had a higher proportion of participants who had previously been initiated on ART (5.1%, *n* = 353) and used HIV testing to re-engage in care, compared to community testing (1.3%, *n* = 7).

### Advanced HIV disease among newly diagnosed HIV patients in community and facility groups

The AHD rate was 19.0% (*n* = 1,248) in the facility group and was 16.7% (*n* = 88) in the community, but the rates were not statistically different between the groups (*p* > 0.05). There were also no statistically significant differences (*p* > 0.05) in AHD rates across any of the measured parameters between community and facility testing locations. In both community and facility testing settings, males had higher AHD rates (community: 21.0%, *n* = 39 vs. facility: 25.1%, *n* = 640; *p* > 0.05). Distribution of AHD by Age groups was also similar; for instance, the AHD rate for the 40–49 age group was 24.9% (*n* = 18) in the community and 26.8% (*n* = 339) in facilities (*p* > 0.05). AHD rates were high among participants who had delayed initiation of 8–28 days regardless of where they were tested (Community: 35.9%, *n* = 14 vs. Facility: 37.7%, *n* = 149; *p* > 0.05) (Table 2).

**Table 2:** Proportion of AHD among participants disaggregated by location of HIV testing.

Parameter	Community	Facility	Total	*P*-value^*^
	*n* (%)	*n* (%)	*n* (%)	
All newly diagnosed HIV clients (N)	526	6,556	7,082	
All participants with AHD	88 (16.7)	1,248 (19.0)	1,336 (18.9)	0.146
Age (years)				
15–29	31 (13.7)	281 (13.4)	312 (13.5)	0.284
30–39	28 (15.1)	456 (20.7)	484 (20.4)	0.113
40–49	18 (24.9)	339 (26.8)	357 (26.7)	0.452
50–59	10 (30.4)	126 (23.8)	136 (24.2)	0.399
60+	1 (9.2)	46 (25.3)	47 (24.4)	0.301
Sex				
Female	49 (14.3)	608 (14.0)	657 (14.1)	0.548
Male	39 (21.0)	640 (25.1)	679 (24.9)	0.097
Marital status				
Widowed/divorced/Separated	2 (22.3)	33 (27.8)	35 (27.4)	0.556
Married	17 (27.5)	294 (21.7)	311 (22.0)	0.231
Single	69 (15.0)	921 (17.1)	990 (17.0)	0.187
Region				
Lubombo	29 (14.4)	373 (15.0)	402 (15.0)	0.635
Manzini	59 (118.0)	875 (20.4)	934 (20.2)	0.233
Smoking				
Yes	2 (20.1)	28 (22.4)	30 (22.2)	0.801
No	86 (16.6)	1,220 (18.8)	1,306 (18.6)	0.142
Alcohol intake				
Yes	5 (16.8)	93 (21.7)	98 (21.4)	0.423
No	83 (16.7)	1,155 (18.6)	1,228 (18.1)	0.174
ART initiation status				
Same day initiation (0–7 days)	64 (14.1)	1,041 (17 0.5)	1,105 (17.3)	0.059
Delayed initiation (8–28 days)	14 (35.9)	149 (37.7)	163 (37.6)	0.822
Delayed initiation (>28 days)	8 (27.6)	42 (23.0)	50 (23.6)	0.585
Pending initiation	2 (66.7)	16 (42.1)	18 (43.9)	0.409

*Compares AHD rates between community and facility.

### Factors associated with AHD in the community and facility setting

As shown in Table 3, in the facility setting, a year increase in age increased the odds of AHD by 2% (aOR: 1.03 [1.01–1.05]; *p* < 0.001) at the time of HIV diagnosis. Males were 1.8 times more likely to present with AHD at the time of HIV diagnosis compared to females (aOR: 1.8 [1.5–2.1]; *p* < 0.001). Conversely, in the community setting, neither age (aOR: 1.02 [0.97–1.06]; *p* > 0.05) nor sex (aOR: 1.2 [0.4–1.5]; *p* > 0.05) were associated with AHD. Among participants tested in the facility setting, residing in the Manzini region increased the odds of having AHD at HIV diagnosis by 60% (aOR: 1.6 [1.3–1.8]; *p* < 0.001) compared to residing in Lubombo. A similar trend was observed in the community setting (aOR: 1.6 [1.0–2.5]) but was not statistically significant (*p* > 0.05). In the community setting, participants with AHD were was associated with delayed ART initiation by 8–28 days (aOR: 3.2 [1.9–4.1]; *p* = 0.002) or delayed ART initiation by >28 days (aOR: 2.7 [1.6–4.2]; *p* = 0.037) compared to those initiated same-day. In the facility setting, AHD was associated with delaying ART initiation by 8–28 days (aOR: 2.7 [2.1–3.3]; *p* < 0.001) or pending ART initiation (aOR: 3.0 [1.4–5.8]; *p* = 0.003) had higher odds of having.

**Table 3:** Factors associated with AHD among community and facility identified newly diagnosed HIV positive clients.

Parameter	Community				Facility			
	cOR	*P*-value	aOR	*P*-value	cOR	*P*-value	aOR	*P*-value
Age (years)	1.03 [1.00–1.06]	0.012	1.02 [0.97–1.06]	0.231	1.04 [1.02–1.06]	<0.001	1.03 [1.01–1.05]	<0.001
Sex								
Female	1							
Male	1.7 [1.1–2.4]	0.037	1.2 [0.4–1.5]	0.201	2.3 [1.9–2.5]	<0.001	1.8 [1.5–2.1]	<0.001
Marital status								
Widowed/divorced/Separated	1							
Married	1.4 [0.2–7.1]	0.621	1.3 [0.2–6.8]	0.754	0.6 [0.4–1.0]	0.137	0.8 [0.51.5]	0.645
Single	0.7 [0.1–3.1]	0.456	0.8 [0.1–4.3]	0.623	0.4 [0.3–0.7]	<0.001	0.7 (0.4–1.2)	0.872
Region								
Lubombo	1							
Manzini	1.5 [0.6–2.3]	0.245	1.6 [1.0–2.5]	0.113	1.6 [1.3–1.8]	<0.001	1.6 [1.3–1.8]	<0.001
Smoking								
Yes	1.4 [0.5–4.6]	0.321	1.1 [0.4–4.1]	0.576	1.4 [0.9–2.0]	0.201	1.1 [0.7–1.3]	0.325
No	1							
Alcohol intake								
Yes	1.1 [0.6–2.1]	0.637	1.1 [0.5–1.8]	0.427	1.3 [1.0–1.6]	0.051	1.0 [0.8–1.3]	0.754
No	1							
ART initiation status								
Same day Initiation (0–7 days)	1							
Delayed Initiation (8–28 days)	3.4 [1.7–6.9]	0.001	3.2 (1.9–4.1)	0.002	0.3 [0.2–0.5]	<0.001	2.7 [2.1–3.3]	<0.001
Delayed Initiation (>28 days)	2.3 [1.0–5.5]	0.053	2.7 [1.6–4.2]	0.037	2.8 [2.3–3.5]	<0.001	1.5 [1.0–2.1]	0.058
Pending Initiation	12.2 [7.0–16.7]	0.042	8.7 [6.1–9.6]	0.062	3.4 [1.8–6.5]	<0.001	3.0 [1.4–5.8]	0.003

## Discussion

Some studies have suggested that Community-based HIV testing, including index testing, is generally more effective at identifying people earlier in their infection compared to facility-based testing, resulting in lower rates of Advanced HIV Disease (AHD). While some studies suggest community-based clients are less likely to have AHD, other findings indicate that for certain settings, facility and community approaches do not differ significantly in identifying AHD. This study aimed to compare the AHD rates between community and facility groups and associated factors in Eswatini. The AHD rate in this study was 19.0% in the facility group and 16.7% in the community group. These rates are lower than the 20–30% observed in sub-Saharan Africa (29, 30) but remain above the global objective of reducing AHD to below 10% among all people entering or re-entering care, a threshold considered indicative of a health system successfully achieving early diagnosis (31). One study in Ethiopia found AHD rates of up to 34.4% among newly diagnosed PLHIV-initiating ART (32). To this end, even though Eswatini surpassed the UNAIDS 95–95–95 epidemic control targets, a gap remains in the quality of the first 95. The AHD rates demonstrated in this study suggest that national-level success can mask sub-groups that continue to enter the care cascade at advanced stages. For instance, in the facility group, males, older individuals, and those in urban residential settings like Manzini had a higher risk of presenting with AHD, marking them as targeted groups for prevention of late presentation (33, 34).

This contrasts the findings in an Ethiopian study that showed that rural residence and being identified through community-based index case testing were found to be independent predictors of AHD (32). Alcohol consumption was also identified as a risk factor (33), contrary to our study findings which did not find a significant association between AHD and alcohol consumption. While our study showed that the facility group had a marginally higher AHD rate than the community group, there was no significant difference detected in this programmatic dataset, but residual confounding and selection into testing modality remain possible. Similar results were reported in a study in rural eastern Uganda which found that facility-based HIV testing services had no significant effect on AHD at diagnosis when compared to community-based HIV testing services (OR 1.20, 95% CI 0.49–2.90) (35). This indicates that community-based testing identifies individuals at similar stages of disease progression as facility-based testing, contrary to the assumption that community testing leads to earlier diagnosis at higher CD4 counts and lower rate of AHD (12, 13). This necessitates an evaluation of the added value of community testing against a backdrop of dwindling global HIV funding. The facility has been identified as the primary entry of late presenters, but this study observed that community testing reached a younger, single population, which is a demographic often reported to bypass facility-based care due to clinic-associated stigma or perceived lower risk (15, 36), yet also known as a mobile, high-transmission group that would otherwise remain undiagnosed until reaching advanced disease stages, potentially increasing secondary transmissions and long-term healthcare costs (37, 38). This implies that both facility and community testing can continue to be implemented as they both produce a similar yield of AHD, and community-based testing can also be used, as a compliment, to target and reach individuals with a higher risk of late presentation and geographic high-burden hubs like Manzini, to address localized drivers of late presentation (39–41).

Our study found that community identified participants were slightly younger on average (mean age 32.9 ± 10.2 years) compared to those identified at facilities (34.7 ± 10.8 years), with a higher proportion of the 15–29 years age band in the community (42.8%, *n* = 228) than in the facility (36.4%, *n* = 2,514) setting. It also found a sex related gap in the diagnosis of AHD, with males being 1.7 times more likely to present with Advanced HIV Disease (AHD) than females, a trend that aligns with global data suggesting that men often delay health-seeking behavior until symptoms become severe (42). This is further compounded by geographic variation, as residents of the Manzini region faced 40% higher odds of AHD at diagnosis compared to those in Lubombo, suggesting regional differences in facility-based screening coverage or health literacy (43), leading to late diagnosis and AHD. Interestingly, the community setting appeared to neutralize these disparities; neither region nor sex were significant predictors of AHD in community-based testing. This corroborates findings from other studies that have suggested that community HIV testing often reaches younger individuals, and that facility HIV testing often identifies older individuals, who are already sick, which can result in a higher proportion of AHD (42, 44).

Delayed ART initiation was significantly associated with AHD across both groups. This association with AHD reflects a challenge where patients with advanced disease often require stabilization of opportunistic infections before beginning ART (45). The implication is that any delay in ART initiation for a patient with AHD, even when indicated for stabilization, requires clinical surveillance and institutional infection-prevention practices managing immunocompromised patients (46) as it represents a period of high mortality risk (1, 30, 47). This finding highlights the need for refined HIV management guidelines that incorporate rapid AHD stabilization pathways to reduce the stabilization window (48) as some clients in this study required >28 days of stabilization. On other note, the study period of this study begins shortly after the acute COVID-19 disruption, which brought changes in testing access, facility attendance, outreach delivery, and linkage-to-care capacity that may have influenced AHD presentation. Some studies have shown that post-emergency hospital-care reorganization work required adaptation of care pathways after SARS-CoV-2 (49), while studies on persistent post-COVID dyspnea and rehabilitation needs highlight how large infectious disease shocks generated longer-term service burden and competing demands on health systems (50). These studies highlight the residual confounding effect of COVID-19 that could have influenced the observed rates of AHD and ART linkage delays in this study. Furthermore, the study observed a higher rate of previously initiated clients returning to care through the facility than community setting, which indicates that facilities remain the central point for re-engagement for PLHIV returning to care (51, 52).

These findings have implications for HIV programs. The finding that AHD remains above the global 10% target despite strong national 95–95–95 performance shows that national cascade success can mask subgroups still presenting late, particularly men, older adults, and clients in Manzini. Community testing did not demonstrate a significant advantage over facility-based testing in early diagnosis of HIV in this context. Hence community testing should not be justified only by presumed earlier diagnosis but by providing a complementary strategy for sub populations who often bypass facility-based care as well as used in addressing localized drivers of late presentation. Facility-based testing, meanwhile, remains essential for symptomatic late presenters and people returning to care.

In conclusion, AHD rates remain above the global 10% threshold, signaling a quality gap in early diagnosis within the care cascade, especially among subgroups that are still presenting late for HIV testing. The rates of AHD are similar across community and facility settings, hence community-based testing does not inherently facilitate earlier diagnosis; however, the community testing can provide a complementary strategic value by targeting late presentation risk groups such as males, older individuals, urban geographic settings and intercepting younger, mobile, and single demographics who often bypass facility-based care, to address localized drivers of late presentation. Furthermore, the association between AHD and delayed ART initiation underscores a need for revised clinical guidelines that promote CD4 testing at facility and community level, ensure that the AHD package is available as soon as possible to eligible individuals, and incorporate rapid AHD stabilization pathways during the pre-treatment window. Future research should focus on the identification of a cost-effective and sustainable model of community testing that will address localized late presentation drivers and improve the quality of the first 95 target in mature epidemics.

### Strengths and limitations

The study used secondary data originally collected for routine clinical and programmatic purposes, which comes with the constraints of real-world health service delivery data such as inherent inconsistencies in documentation, missing variables, or lack of standardized clinical staging across different sites. The use of programmatic records limited the exploration of all the important variables as the study was limited to the variables already available in the electronic medical records, preventing the inclusion of nuanced socio-behavioral factors such as barriers to testing, socio-economic status, educational level, access to care, and distance to facility or stigma that could further explain the factors associated with AHD. While the two groups had no evidence statistical differences in characteristic distribution of participants and AHD rates, community group had much fewer participants which could have affected the statistical power in multivariable analysis in the community group, leading to statistically non-significant characteristics. We applied population sampling to include all available eligible participants in the analysis, and each group was analyzed separately in regression analysis to identify factors associated with AHD in each group, hence avoiding the risk of having the larger facility group overshadow the smaller community group. Furthermore, the cross-sectional nature of the study captures only a snapshot in time at the point of diagnosis, which may lead to an underestimation of AHD burden if clinical symptoms were not fully documented at the initial visit. Despite these limitations, the use of a large, population-level programmatic dataset provides high ecological validity, reflecting the actual performance of Eswatini’s HIV response in routine care settings.

## Data Availability

The original contributions presented in the study are included in the article/supplementary material, further inquiries can be directed to the corresponding author.

## References

[1] World Health Organization (WHO). (2025). WHO Guidelines on the Management of Advanced HIV Disease. Geneva, Switzerland: WHO. Available online at: https://iris.who.int/server/api/core/bitstreams/68dfe26f-ad54-4f60-b92f-b943b1a0d82c/content (Accessed November 5, 2025).

[2] Ford N Kassanjee R Stelzle D Jarvis JN Sued O Perrin G . Global prevalence of advanced HIV disease in healthcare settings: a rapid review. J Int AIDS Soc. (2025) 28:e26415. doi: 10.1002/jia2.26415, 39915008 PMC11802239

[3] Ford N Meintjes G Calmy A Bygrave H Migone C Vitoria M . Managing advanced HIV disease in a public health approach. Clin Infect Dis. (2018) 66:S106–10. doi: 10.1093/cid/cix1139, 29514232 PMC5850613

[4] Gupta RK Gregson J Parkin N Haile-Selassie H Tanuri A Andrade Forero L . HIV-1 drug resistance before initiation or re-initiation of first-line antiretroviral therapy in low-income and middle-income countries: a systematic review and meta-regression analysis. Lancet Infect Dis. (2018) 18:346–55. doi: 10.1016/s1473-3099(17)30702-8, 29198909 PMC5835664

[5] Haumba S Arora S Williams V Maseko T Mafukidze A Ojoo S. Prevalence, predictors, and outcomes of HIV care in HIV-positive clients entering HIV care with advanced HIV disease in sub-Saharan Africa 2010–2022: systematic review and meta-analysis. Health Sci Report. (2024) 7:e70285. doi: 10.1002/hsr2.70285, 39720238 PMC11667100

[6] Eswatini Ministry of Health. Annual HIV Program Report 2022/2023. Mbabane, Eswatini: Ministry of Health, Eswatini (2023).

[7] Johnson LF van Rensburg C Govathson C Meyer-Rath G. Optimal HIV testing strategies for South Africa: a model-based evaluation of population-level impact and cost-effectiveness. Sci Rep. (2019) 9:12621. doi: 10.1038/s41598-019-49109-w, 31477764 PMC6718403

[8] Chihana M Conan N Ohler L Huerga H Wanjala S Masiku C . Changes over time in the proportion of advanced HIV disease in two high HIV prevalence settings in Ndhiwa (Kenya) and Eshowe (South Africa). J Int Assoc Provid AIDS Care. (2024) 23:23259582241260219. doi: 10.1177/23259582241260219, 38881294 PMC11185002

[9] Chihana ML Huerga H Van Cutsem G Ellman T Goemaere E Wanjala S . Distribution of advanced HIV disease from three high HIV prevalence settings in sub-Saharan Africa: a secondary analysis data from three population-based cross-sectional surveys in Eshowe (South Africa), Ndhiwa (Kenya) and Chiradzulu (Malawi). Glob Health Action. (2019) 12:1679472. doi: 10.1080/16549716.2019.1679472, 31679482 PMC6844432

[10] Chamie G Napierala S Agot K Thirumurthy H. HIV testing approaches to reach the first UNAIDS 95% target in sub-Saharan Africa. Lancet HIV. (2021) 8:e225–36. doi: 10.1016/s2352-3018(21)00023-0, 33794183

[11] Joint United Nations Programme on HIV/AIDS (UNAIDS). (2023). Harnessing Community Models for HIV Testing. Geneva, Switzerland: UNAIDS. Available online at: https://thepath.unaids.org/ (Accessed November 5, 2025).

[12] Lamp K McGovern S Fong Y Atem CD Nfetam JBE Nzuobontane D . Proportions of CD4 test results indicating advanced HIV disease remain consistently high at primary health care facilities across four high HIV burden countries. PLoS One. (2020) 15:e0226987. doi: 10.1371/journal.pone.0226987, 31910221 PMC6946176

[13] Asare K Lewis L van der Molen J Sookrajh Y Khubone T Ngwenya T . Impact of increasing CD4 count threshold eligibility for antiretroviral therapy initiation on advanced HIV disease and tuberculosis prevalence and incidence in South Africa: an interrupted time series analysis. BMJ Glob Health. (2025) 10:e016631. doi: 10.1136/bmjgh-2024-016631, 40204463 PMC11987148

[14] Stelzle D Rangaraj A Jarvis JN Razakasoa NH Perrin G Low-Beer D . Prevalence of advanced HIV disease in sub-Saharan Africa: a multi-country analysis of nationally representative household surveys. Lancet Glob Health. (2025) 13:e437–46. doi: 10.1016/S2214-109X(24)00538-2, 40021302 PMC11868778

[15] Eshun-Wilson I Awotiwon AA Germann A Amankwaa SA Ford N Schwartz S . Effects of community-based antiretroviral therapy initiation models on HIV treatment outcomes: a systematic review and meta-analysis. PLoS Med. (2021) 18:e1003646. doi: 10.1371/journal.pmed.1003646, 34048443 PMC8213195

[16] Dovel K Shaba F Offorjebe OA Balakasi K Nyirenda M Phiri K . Effect of facility-based HIV self-testing on uptake of testing among outpatients in Malawi: a cluster-randomised trial. Lancet Glob Health. (2020) 8:e276–87. doi: 10.1016/S2214-109X(19)30534-0, 31981557

[17] Sileo KM Fielding-Miller R Dworkin SL Fleming PJ. A scoping review on the role of masculine norms in men’s engagement in the HIV care continuum in sub-Saharan Africa. AIDS Care. (2019) 31:1435–46. doi: 10.1080/09540121.2019.1595509, 30909724 PMC10512737

[18] Bendera A Baryomuntebe D Kevin NW Nanyingi M Kinengyere P Mujeeb S . Determinants of late HIV diagnosis and advanced HIV disease among people living with HIV in Tanzania. HIV-AIDS Res Palliat Care. (2024) 16:313–23. doi: 10.2147/hiv.s473291, 39220740 PMC11363941

[19] Cornell M Majola M Johnson LF Dubula-Majola V. HIV services in sub-Saharan Africa: the greatest gap is men. Lancet. (2021) 397:2130–2. doi: 10.1016/s0140-6736(21)01163-6, 34087108

[20] Fomundam HN Tesfay AR Mushipe SA Mosina MB Boshielo CT Nyambi HT . Prevalence and predictors of late presentation for HIV care in South Africa. S Afr Med J. (2017) 107:1058–64. doi: 10.7196/SAMJ.2017.v107i12.12358, 29262956 PMC5792655

[21] Kerschberger B Schomaker M Kuwengwa R Ciglenecki I Pasipamando J Mthethwa-Hleza S . The impact of same-day antiretroviral therapy initiation under the World Health Organization treat-all policy. Am J Epidemiol. (2021) 190:1519–32. doi: 10.1093/aje/kwab032 33576383 PMC8327202

[22] Hakim J Musiime V Szubert AJ Mallewa J Siika A Agutu C . Enhanced prophylaxis plus antiretroviral therapy for advanced HIV infection in Africa. N Engl J Med. (2017) 377:233–45. doi: 10.1056/nejmoa1615822, 28723333 PMC5603269

[23] Maughan-Brown B Harrison A Galárraga O Kuo C Smith P Bekker LG . Factors affecting linkage to HIV care and ART initiation following referral for ART by a mobile health clinic in South Africa: evidence from a multimethod study. J Behav Med. (2019) 42:883–97. doi: 10.1007/s10865-018-0005-x, 30635862 PMC6625943

[24] Maughan-Brown B Beckett S Kharsany ABM Cawood C Khanyile D Lewis L . Poor rates of linkage to HIV care and uptake of treatment after home-based HIV testing among newly diagnosed 15-to-49-year-old men and women in a high HIV prevalence setting in South Africa. AIDS Care. (2021) 33:70–9. doi: 10.1080/09540121.2020.1719025, 32036678 PMC7477751

[25] Nabukeera S Kagaayi J Makumbi FE Mugerwa H Matovu JKB Oliver D . Factors associated with virological non-suppression among HIV-positive children receiving antiretroviral therapy at the joint clinical research Centre in Lubowa, Kampala Uganda. PLoS One. (2021) 16:e0246140. doi: 10.1371/journal.pone.0246140 33503074 PMC7840004

[26] Eswatini Ministry of Health (MOH). Eswatini Integrated HIV Management. Guidelines Government of Eswatini. Mbabane, Eswatini: Ministry of Health, Eswatini (2022). Available online at: http://swaziaidsprogram.org/wp-content/uploads/2023/08/Eswatini-Integrated-HIV-Management-Guidelines-1.pdf (Accessed November 6, 2025).

[27] Vojnov L Markby J Boeke C Harris L Ford N Peter T. POC CD4 testing improves linkage to HIV care and timeliness of ART initiation in a public health approach: a systematic review and meta-analysis. PLoS One. (2016) 11:e0155256. doi: 10.1371/journal.pone.0155256, 27175484 PMC4866695

[28] Central Statistics Office. Government of Eswatini. 2017–2038 POPULATION PROJECTION – Based on THE 2017 Eswatini Population and Housing Census – AUGUST 2020. Mbabane, Eswatini: Ministry of Economic Planning and Development, Eswatini. (2020). Available online at: https://www.gov.sz/images/Final-2017_2038-POPULATION-PROJECTIONS-1-1.pdf (Accessed November 8, 2025).

[29] Carmona S Bor J Nattey C Maughan-Brown B Maskew M Fox MP . Persistent high burden of advanced HIV disease among patients seeking care in South Africa’s national HIV program: data from a nationwide laboratory cohort. Clin Infect Dis. (2018) 66:S111–7. doi: 10.1093/cid/ciy045, 29514238 PMC5850436

[30] Patten GE Euvrard J Anderegg N Boulle A Arendse KD von der Heyden E . Advanced HIV disease and engagement in care among patients on antiretroviral therapy in South Africa: results from a multi-state model. AIDS. (2023) 37:513–22. doi: 10.1097/QAD.0000000000003442, 36695361 PMC9881824

[31] UNAIDS. Global AIDS Strategy 2021–2026: End Inequalities, End AIDS. Geneva, Switzerland; (2021). Available online at: https://www.unaids.org/en/Global-AIDS-Strategy-2021-2026 (Accessed January 8, 2026).

[32] Lerango TL Markos T Yehualeshet D Kefyalew E Lerango SL. Advanced HIV disease and its predictors among newly diagnosed PLHIV in the Gedeo zone, southern Ethiopia. PLoS One. (2024) 19:e0310373. doi: 10.1371/journal.pone.0310373, 39269935 PMC11398689

[33] Fuge TG Tsourtos G Miller ER. Risk factors for late linkage to care and delayed antiretroviral therapy initiation among adults with HIV in sub-Saharan Africa: a systematic review and meta-analysis. Int J Infect Dis. (2022) 122:885–904. doi: 10.1016/j.ijid.2022.07.037, 35843499

[34] Smit M Brinkman K Geerlings S Smit C Thyagarajan K van Sighem A . Future challenges for clinical care of an ageing population infected with HIV: a modelling study. Lancet Infect Dis. (2015) 15:810–8. doi: 10.1016/S1473-3099(15)00056-0, 26070969 PMC4528076

[35] Izudi J Ssentongo SM Appeli S Bajunirwe F. Effect of facility- versus community-based HIV testing services on the diagnosis of advanced HIV disease in Uganda: a quasi-experimental study. BMC Infect Dis. (2025) 26:223. doi: 10.1186/s12879-025-12488-9, 41469583 PMC12860039

[36] Pantelic M Casale M Cluver L Toska E Moshabela M. Multiple forms of discrimination and internalized stigma compromise retention in HIV care among adolescents: findings from a south African cohort. J Int AIDS Soc. (2020) 23:e25488. doi: 10.1002/jia2.25488, 32438498 PMC7242009

[37] Garland JM Mayan H Kantor R. Treatment of advanced HIV in the modern era. Drugs. (2025) 85:883–909. doi: 10.1007/s40265-025-02181-1, 40354016 PMC12277976

[38] Chamie G Clark TD Kabami J Kadede K Ssemmondo E Steinfeld R . A hybrid mobile approach for population-wide HIV testing in rural East Africa: an observational study. Lancet HIV. (2016) 3:e111–9. doi: 10.1016/S2352-3018(15)00251-9, 26939734 PMC4780220

[39] Groves AK Stankard P Bowler SL Jamil MS Gebrekristos LT Smith PD . A systematic review and meta-analysis of the evidence for community-based HIV testing on men’s engagement in the HIV care cascade. Int J STD AIDS. (2022) 33:1090–105. doi: 10.1177/09564624221111277, 35786140 PMC9660288

[40] Haas AD Radin E Birhanu S Low AJ Saito S Sachathep K . Prevalence of and factors associated with late diagnosis of HIV in Malawi, Zambia, and Zimbabwe: results from population-based nationally representative surveys. PLOS Global Public Health. (2022) 2:e0000080. doi: 10.1371/journal.pgph.0000080, 36962254 PMC10021857

[41] Hlongwa M Mashamba-Thompson T Makhunga S Hlongwana K. Barriers to HIV testing uptake among men in sub-Saharan Africa: a scoping review. Afr J AIDS Res. (2020) 19:13–23. doi: 10.2989/16085906.2020.1725071, 32174231

[42] Sharma M Ying R Tarr G Barnabas R. Systematic review and meta-analysis of community and facility-based HIV testing to address linkage to care gaps in sub-Saharan Africa. Nature. (2015) 528:S77–85. doi: 10.1038/nature16044, 26633769 PMC4778960

[43] Eswatini Ministry of Health. Annual Health Statistics Report 2023. Mbabane, Eswatini: Eswatini Government (2023).

[44] Suthar AB Ford N Bachanas PJ Wong VJ Rajan JS Saltzman AK . Towards universal voluntary HIV testing and counselling: a systematic review and meta-analysis of community-based approaches. PLoS Med. (2013) 10:e1001496. doi: 10.1371/journal.pmed.1001496, 23966838 PMC3742447

[45] Brust J. C. M. McGowan J. P. Fine S. M. Smith A. B. Jones C. D. White E. F. . (2024). Management of immune reconstitution inflammatory syndrome (IRIS). In: NCBI Bookshelf. Baltimore (MD): Johns Hopkins University. Available online at: https://www.ncbi.nlm.nih.gov/books/NBK570544/ (Accessed January 23, 2026).

[46] Lattanzio S Cuscianna E Riformato G Noviello C Tafuri S Bianchi FP. Human and environmental impact of chemical agents used for cleaning, antisepsis, disinfection and sterilization in healthcare facilities. A semi-qualitative study among medical institutions of Puglia and Basilicata, southern Italy. Acta Biomed. (2025) 96:e202516146. doi: 10.23750/abm.v96i6.16146

[47] Tenforde MW Gertz AM Lawrence DS Wills NK Guthrie BL Farquhar C . Mortality from HIV-associated meningitis in sub-Saharan Africa: a systematic review and meta-analysis. J Int AIDS Soc. (2020) 23:e25416. doi: 10.1002/jia2.25416, 31957332 PMC6970088

[48] Tao Y Xiao X Zhang C Xie Y Wang H. Prevalence of delayed antiretroviral therapy initiation among people living with HIV: a systematic review and meta-analysis. PLoS One. (2023) 18:e0286476. doi: 10.1371/journal.pone.0286476, 37874794 PMC10597480

[49] Bianchi FP Cuscianna E Di Lorenzo A Daleno A Mele F Marra M . A new paradigm of hospital care for SARS-COV-2 patients in the post-emergency phase in Italy. Ann Ig. (2023) 35:250–3. doi: 10.7416/ai.2022.2546, 36222605

[50] Resta E Quarato CMI Scioscia G Cuscianna E Tondo P Mansueto G . Low-intensity rehabilitation in persistent post COVID-19 dyspnoea: the value of Spa health resort as appropriate setting. Ann Ig. (2024) 36:597–613. doi: 10.7416/ai.2024.2617, 38436080

[51] Mirzazadeh A Eshun-Wilson I Thompson RR Bonyani A Kahn JG Baral SD . Interventions to reengage people living with HIV who are lost to follow-up from HIV treatment programs: a systematic review and meta-analysis. PLoS Med. (2022) 19:e1003940. doi: 10.1371/journal.pmed.1003940, 35290369 PMC8923443

[52] Arendse KD Walker C Pfaff C Lebelo K Cassidy T Isaakidis P . Supporting re-engagement with HIV services after treatment interruption in South Africa: a mixed method program evaluation of MSF’S welcome service. Sci Rep. (2024) 14:7317. doi: 10.1038/s41598-024-57774-9, 38538754 PMC10973441

